# The Frequency of Occurrence of Resistance and Genes Involved in the Process of Adhesion and Accumulation of Biofilm in *Staphylococcus aureus* Strains Isolated from Tracheostomy Tubes

**DOI:** 10.3390/microorganisms10061210

**Published:** 2022-06-14

**Authors:** Kamil Drożdż, Dorota Ochońska, Łukasz Ścibik, Monika Gołda-Cępa, Katarzyna Biegun, Monika Brzychczy-Włoch

**Affiliations:** 1 Department of Molecular Medical Microbiology, Chair of Microbiology, Faculty of Medicine, Jagiellonian University Medical College, 31-121 Krakow, Poland; dorota.ochonska@uj.edu.pl (D.O.); l.scibik@5wszk.com.pl (Ł.Ś.); k.biegun@uj.edu.pl (K.B.); 2 Department of Otolaryngology, Head and Neck Surgery, 5th Military Hospital with Polyclinic, 30-090 Krakow, Poland; 3 Faculty of Chemistry, Jagiellonian University, 31-007 Krakow, Poland; mm.golda@uj.edu.pl

**Keywords:** *Staphylococcus aureus*, PFGE, antibiotic resistance, tracheostomy tube, biofilm, MSCRAMMs

## Abstract

**Background**: Bacterial biofilm on the surface of tracheostomy tubes (TTs) is a potential reservoir of potentially pathogenic bacteria, including *S. aureus*. For this reason, our study aimed to investigate biofilm production in vitro and the presence of *ica*AD and MSCRAMM genes in clinical *S. aureus* strains derived from TTs, with respect to antibiotic resistance and genetic variability. **Methods:** The clonality of the *S. aureus* strains was analyzed by the PFGE method. The assessment of drug resistance was based on the EUCAST recommendations. The isolates were evaluated for biofilm production by the microtiter plate method and the slime-forming ability was tested on Congo red agar (CRA). The presence of *ica*AD genes was investigated by PCR and MSCRAMM genes were detected by multiplex PCR. **Results:** A total of 60 patients were enrolled in the study. One TT was obtained from each patient (n = 60). Twenty-one TTs (35%) were colonized with S. aureus. A total of 24 strains were isolated as 3 patients showed colonization with 2 SA clones (as confirmed by PFGE). PFGE showed twenty-two unique molecular profiles. Two isolates (8%) turned out to be MRSA, but 50% were resistant to chloramphenicol, 25% to erythromycin and 8% to clindamycin (two cMLS_B_ and four iMLS_B_ phenotypes were detected). The microtiter plate method with crystal violet confirmed that 96% of the strains were biofilm formers. Representative strains were visualized by SEM. All isolates had *clf*AB, *fnb*A, *ebp*S and *ica*AD. Different MSCRAMM gene combinations were observed. **Conclusions:** the present study showed that the *S. aureus* isolated from the TTs has a high diversity of genotypes, a high level of antibiotic resistance and ability to produce biofilm.

## 1. Introduction

*Staphylococcus aureus* is a common opportunistic organism that colonizes human skin, the area from the front of the anterior nasal vestibule to the back of the nasopharynx [1,2]. *S. aureus* is a major human pathogen causing many clinical infections. It can cause bacteremia, infectious endocarditis, osteoarthritis, skin and soft tissue infections, pleuritis and lung infections, metastatic abscesses, sepsis and toxic shock syndrome [3].

An important group of *S. aureus* infections are biomaterial-associated infections (BAIs) resulting from commonly used medical devices, including tracheostomy tubes (TTs) made of various biomaterials. For their production, polyvinyl chloride and polyethylene are most often used [4]. Very often, BAIs are a consequence of biomaterial colonization [5,6,7,8]. Patients with tracheostomy are colonized with a variety of microbiota, represented by various species of Gram-positive and Gram-negative bacteria [5,7]. Improper patient care and too infrequent replacement of tracheostomy tubes may lead to colonization and then translocation of microorganisms to the lower parts of the respiratory tract, initiating the inflammatory process [5,7].

According to the current literature review, among Gram-positive bacteria, *S. aureus* is a common microorganism that colonizes tracheostomy tubes and an etiological agent in the pathogenesis of respiratory tract infections in hospitalized patients undergoing tracheotomy surgery [5,7,8,9].

In 2002, the National Institutes of Health (NIH) estimated that over 80% of microbial infections in the human body are associated with the formation of biofilm [5]. The ability of *S. aureus* to form a biofilm on the surface of biomaterials, which are widely used in medicine, plays a key role and is considered one of the main factors of virulence [10,11,12,13,14].

An important determinant of the adhesive capacity of *S. aureus* is the large family of MSCRAMM (microbial surface components recognizing adhesive matrix molecules) surface proteins. These proteins are anchored in the cell wall peptidoglycan by sortase A and have multiple signaling domains, thanks to which the family is involved in the initial stages of biofilm formation. MSCRAMMs are able to attach to many components of the host extracellular matrix (ECM) and blood plasma, including to collagen, fibronectin, elastin and many others that cover the surface of biomaterials [15]. In the early stages of biofilm formation, the expression of these proteins is increased. Along with the subsequent stages of maturation, there is a gradual reduction in the expression of MSCRAMM proteins and an increased share of polysaccharide intercellular adhesins (PIA), which facilitate the aggregation of bacterial cells. PIA is a linear homoglycan containing at least 130 β-1,6 linked N-acetylglucosamine residues, 15–20% of which are deacetylated [16]. Deacetylation confers an electropositive nature to normally neutral molecules. This charge promotes the aggregation of negatively charged staphylococci. The genes responsible for the production of PIA are located on the *ica* operon, the most important of which is the *ica*A gene, which encodes the enzyme responsible for the formation of N-acetylglucosamine oligomers, with relatively low expression. The activity of this gene is enhanced by the presence of the *ica*D gene [17]. Bacterial cells coated with intercellular polysaccharide adhesins are much more resistant to the mechanisms of the host immune system and the action of antibiotics.

Due to the lack of available literature data on *S. aureus* strains isolated from TTs from patients with tracheostomy, there is an urgent need to supplement these studies with missing data. Therefore, in this study, we aimed at characterizing *S. aureus* strains isolated from tracheostomy tubes, taking into account drug resistance, the ability to create biofilm, and genes from the MSCRAMM family involved in the adhesion process.

## 2. Materials and Methods

### 2.1. Bacterial Strains

Swabs of external and internal surfaces of tracheostomy tubes were collected from 60 tracheostomy tubes from individual patients admitted to the 5th Military Hospital with Polyclinic in Krakow, located in the Malopolska region (Poland). The strains were collected from June 2019 to May 2021. All isolates were placed into the Microbank™ system (Pro Lab Diagnostics Inc., Richmond Hill, ON, Canada) and stored at −80 °C for further analyses. The following four reference strains were used: *S. aureus* ATCC^®^ 25923™ and *S. epidermidis* ATCC^®^ 35984™ (RP62A) known for their slime production, *S. epidermidis* ATCC^®^ 12228™ known as non-slime-producing, and *S. aureus* ATCC^®^ 29213™ as a quality control in antimicrobial susceptibility testing.

### 2.2. Phenotypic Identification of Staphylococcus aureus

The tracheal swabs examined were plated on agar supplemented with 5% sheep blood and later incubated overnight at 37 °C. Each culture underwent Gram staining and was identified to species using basic microbiological techniques, such as catalase test, tube coagulase tests, clumping factor and API Staph tests (bioMérieux) [18].

### 2.3. Antimicrobial Susceptibility Testing

Drug resistance of the *S. aureus* was determined in accordance with the latest EUCAST recommendations, version 11.0, 1 January 2021 [19].

Drug resistance was determined by the Kirby–Bauer disc diffusion method (Oxoid) for amikacin (30 µg), clindamycin (2 µg), erythromycin (15 µg), and fusidic acid (10 µg). 

The E-test method, determining the MIC (minimal inhibitory concentration) value with the use of concentration gradient strips (Liofilchem MIC Test Strips), was used for chloramphenicol (0.016–256 mg/L), linezolid (0.016–256 mg/L), gentamicin (0.016–256 mg/L), tetracycline (0.016–256 mg/L), tobramycin (0.016–256 mg/L), trimethoprim/sulfamethoxazole (0.002–32 mg/L) and vancomycin/teicoplanin (0.008–128 mg/L). If the strains showed resistance to norfloxacin (0.016–256 mg/L), an additional test for ciprofloxacin (0.002–32 mg/L), levofloxacin (0.002–32 mg/L), moxifloxacin (0.002–32 mg/L) and ofloxacin (0.002–32 mg/L) was performed. *S. aureus* ATCC^®^ 29213™ was used as a reference strain.

### 2.4. Genomic DNA Extraction

Genomic DNA was isolated from *S. aureus* strains using the GeneMATRIX Bacterial & Yeast Genomic DNA Purification Kit (EURX) according to the manufacturer’s protocol. The concentration and purity of the isolated DNA were assessed using a NanoDrop Lite spectrophotometer (Thermo Fisher Scientific, Waltham, MA, USA).

### 2.5. Pulsed Field Gel Electrophoresis (PFGE)

The PFGE method was used to determine the genetic relationship among the investigated *S. aureus* isolates, following the previously described method with modifications [20]. The electrophoresis was performed using a CHEF-DR^®^ II PFGE apparatus (Bio-Rad, Hercules, CA, USA).

The similarity of the PFGE profiles was compared according to the percentage similarity of the profiles estimated by the Dice coefficient and grouped by UPGMA (unweighted pair group arithmetic mean method) using GelCompar II software version 6.5 (Applied Maths). The analysis used *S. aureus* ATCC^®^ 25923™ as a reference strain.

### 2.6. Molecular Confirmation of the Species Staphylococcus aureus

The bacterial isolates, which were identified to be *S. aureus* by specific conventional phenotypic methods, were further tested by multiplex PCR with the use of specific primers synthesized by Genomed [18]. 

### 2.7. Methicillin-Resistant Staphylococcus aureus Screening

Methicillin-resistant *S. aureus* (MRSA) were determined using cefoxitin discs (30 µg) on Mueller–Hinton agar (bioMérieux, Marcy-l’Étoile, France) plates in accordance with the European Committee on Antimicrobial Susceptibility Testing [19] Breakpoint tables, version 11.0, 1 January 2021. Isolates with phenotypic resistance to oxacillin (1 µg) were later confirmed to harbor the *mec*A gene by multiplex PCR with the use of specific primers synthesized by Genomed [18].

### 2.8. Quantification of Biofilm in Microtiter Plates

The quantitative assessment of biofilm production by the tested *S. aureus* isolates was based on the published literature data [21], with some modifications. Several identical looking colonies were picked from agar media and then suspended in 8 mL of Tryptic Soy Broth (TSB, Becton Dickinson, Franklin Lakes, NJ, USA) to obtain a 0.5 McFarland inoculum. The wells were inoculated with a suspension of 2 mL of bacteria in sterile polystyrene 12-well flat bottom microtiter plates (Costar^®^ Corning). Incubation was carried out at 37 °C for 18 h. After this time, the culture medium was very gently removed with a pipette, and the sealed bacterial cells were washed three times with PBS and fixed with 2 mL of methanol (POCH S.A., Gliwice, Poland) for 30 s and allowed to dry at 37 °C for 2 h. Thereafter, the wells were stained with crystal violet (ANALAB) for 15 min, then the stain was rinsed with distilled water, and the plates were allowed to dry at 37 °C for 4 h. The negative controls were wells filled with sterile TSB medium (Becton Dickinson). The experiment was performed in triplicate. The absorbance was measured spectrophotometrically at a wavelength of λ = 570 nm in an Infinite 200 Pro (TECAN) instrument supported by the i-control 2.0.10.0 software. Readings in the wells were made on a square-shaped grid with dimensions of 15 × 15. Mean values (OD) and standard deviation (SD) values were calculated for all replicates of the experiment. The cut-off point (ODc) was calculated according to the following formula: ODc = average OD of negative control + (3 × standard deviation (SD) of negative control). The estimated OD value of the tested strains was reduced by the ODc value. The ODc value was determined separately for each 12-well plate separately. The results were interpreted in accordance with the following definitions proposed by Stepanović et al.: OD ≤ ODc is a no biofilm producer; ODc < OD ≤ 2xODc is a weak biofilm producer; 2xODc < OD ≤ 4xODc is a moderate biofilm producer; 4xODc < OD is a strong biofilm producer [22].

### 2.9. Phenotypic Characterization of Slime Producing Ability on Congo Red Agar (CRA)

The slime production assay was performed by cultivation of the *S. aureus* strains on Congo red agar (CRA) plates, as described by Freeman et al. [23]. The plates were prepared by adding 0.8 g Congo red (Sigma, St. Louis, MO, USA) and 36 g of sucrose (Sigma, St. Louis, MO, USA) to 1 L of brain heart infusion agar (BHI, Oxoid, Basingstoke, UK). The inoculated CRA plates were incubated at 37 °C for 24 h. Slime-producing strains develop black colonies, whereas non-producing isolates form red colonies. In the study, a six-point scale developed by Arciola et al. was used to assess the color of the colonies produced. Very black (vb) and black colonies (b) were considered as normal slime producing strains, while almost black (ab) colors were considered as indicative of a weak slime production activity. On the other hand, bordeaux (brd), red ®, and very red (vr) were classified as strains unable to produce slime [24].

### 2.10. MSCRAMM Gene Detection

Multiplex PCR (mPCR) with the use of specific primers (Genomed), as published by Tristan et al. was used to detect the presence of genes encoding microbial surface components that recognize adhesive matrix molecules (MSCRAMMs), including *bbp* (encoding bone sialoprotein binding protein), *clf*A and *clf*B (encoding clumping factors A and B), *cna* (encoding collagen binding protein), *ebp*S (encoding elastin binding protein), *eno* (encoding laminin binding protein), *fnb*A (encoding fibronectin binding protein A), *fnb*B (encoding fibronectin binding protein B) and *fib* (encoding fibrinogen binding protein) (Table 1) [25]. The amplification reaction was performed using the PCR Mix Plus kit (A&A Biotechnology, Gdańsk, Poland).

### 2.11. icaAD Gene Detection

Two PCR assays using specific primers (Genomed) were used to detect *ica*A and *ica*D genes, as proposed by Piechota et al. [11] (Table 1). The amplification reaction was performed using the PCR Mix Plus kit (A&A Biotechnology).

### 2.12. Scanning Electron Microscopy (SEM)

The polyethylene (DEMED) and polyvinyl chloride (SUMI) tracheostomy tubes were aseptically cut into approximately 1 cm fragments and incubated in 4 mL of the 0.5 MacFarland bacterial inoculum in 12-well plates (Costar^®^ Corning). For this purpose, one strain (TT_5) showing a large biofilm production was selected and it was incubated with TT fragments in static culture at 37 °C for 24 h in an aerobic atmosphere. The samples were fixed according to the procedure by Pajerski et al. [26]. The dry samples were glued with carbon tape and carbon glue to an aluminum table and dusted with gold for 1 min (Quorum Q150T S). Observation of the biofilm was performed using a HITACHI S-4700 microscope with the NORAN Vantage microanalysis system.

## 3. Results

### 3.1. Characteristics of Patients

Patients from whom tracheostomy tubes were obtained were hospitalized at the 5th Military Hospital with Polyclinic in Krakow in 2019–2021 in the Department of Otolaryngology (83%, n = 50) due to laryngeal cancer (73%, n = 44), tonsil cancer (3%, n = 2), tongue cancer (2%, n = 1), retromolar trigone cancer (3%, n = 2), cervical abscess (2%, n = 1), as well as in the intensive care unit (17%, n = 10) due to respiratory failure (17%, n = 10). In total, tubes were obtained from 60 people, including 20% (n = 12) of women and 80% (n = 48) of men. The mean age of the people was 67.9 ± 11.9, while in the group of women, it was 77.7 ± 10.2 and in the group of men, 65.5 ± 11.2. Tracheostomy tubes in the ORL department were used for one day, while in the ICU department, they were used for 11.6 ± 6.6 days, on average.

### 3.2. Bacterial Strains and MRSA Screening

A total of 24 isolates were detected on the tested TTs. Using phenotypic and molecular methods, it was confirmed that they belong to the species *S. aureus*, which was detected in 37% (n = 22) of tracheostomy tubes. Only two strains (8%) showed resistance to oxacillin and cefoxitin and possessed the *mec*A gene (MRSA). The remaining 22 strains (92%) showed phenotypic sensitivity to oxacycline and cefoxitin and lacked the *mec*A gene (MSSA).

### 3.3. Antimicrobial Susceptibility Testing 

**Figure 1:**
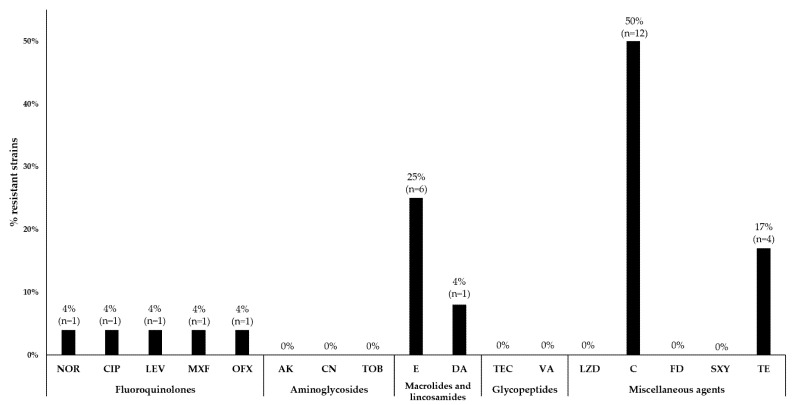
Percentage of *S. aureus* resistance to specific antibiotics. Abbreviations: NOR—norfloxacin; CIP—ciprofloxacin; LEV—levofloxacin; MXF—moxifloxacin; OFX—ofloxacin; AK—amikacin; CN—gentamicin; TOB—tobramycin; E—erythromycin; DA—clindamycin; TEC—teicoplanin; VA—vancomycin; LZD—linezolid; C—chloramphenicol; FD—fusidic acid; SXY—trimethoprim/sulfamethoxazole; TE–tetracycline.

In the tested samples, a high number of strains showed resistance to the selected antibiotics. The highest percentage was represented by strains resistant to chloramphenicol (50%, n = 12), while 25% (n = 6) were resistant to erythromycin, and 8% (n = 2) were resistant to clindamycin. Among these strains, two isolates with the constitutive MLS_B_ (cMLS_B_) phenotype and four with the inducible MLS_B_ (iMLS_B_) were detected. Additionally, 17% of the observed strains were resistant to tetracycline (n = 4). One strain was resistant to norfloxacin (4%), which also showed resistance to ciprofloxacin, levofloxacin, moxifloxacin and ofloxacin. Strains resistant to aminoglycosides (amikacin, gentamicin, tobramycin), glycopeptides (vancomycin and teicoplanin) or other antibiotics, such as linezolid, fusidic acid and trimethoprim with sulfamethoxazole, were not observed (Figure 1).

### 3.4. PFGE

**Figure 2:**
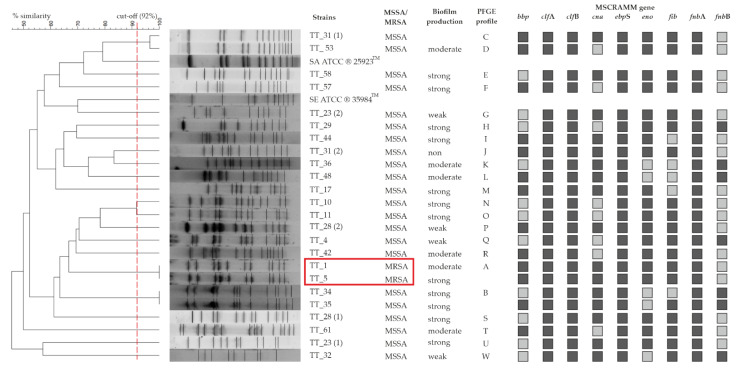
Macrorestriction analysis of the chromosomal DNA from *S. aureus* isolated from tracheostomy tubes after digestion with the restriction enzyme *Sma*I. Dendrogram resulting from computer-assisted analysis of the PFGE profiles (GelCompar II, Dice, UPGMA, 1% optimization, 1% tolerance). LEGEND: the MRSA strains (clone A) are marked with a red box, while the cut-off point (92%) is marked with the red dashed line. A dark gray square indicates the presence of the MSCRAMM family gene, and a light gray square indicates no gene.

The study analyzed the similarity of *Sma*I PFGE restriction profiles for 24 *S. aureus* strains isolated from tracheostomy tube swabs. Among the tested strains, 22 PFGE genotypes (designated from A to W) were identified, 2 of which were distinguished (A and B) because each of them was represented by 2 tested *S. aureus* isolates with a similarity of 100%. The remaining 20 strains showed unique restriction patterns (singletons), with similar PFGE profiles ranging from 49.1% to 91.4%. Additionally, three TTs were colonized by two genetically different strains of *S. aureus*. The dendrogram generated by the computer assisted analysis of the PFGE profiles showed great genetic variation in the isolates (Figure 2).

### 3.5. Quantification of Biofilm in Microtiter Plates

**Figure 3:**
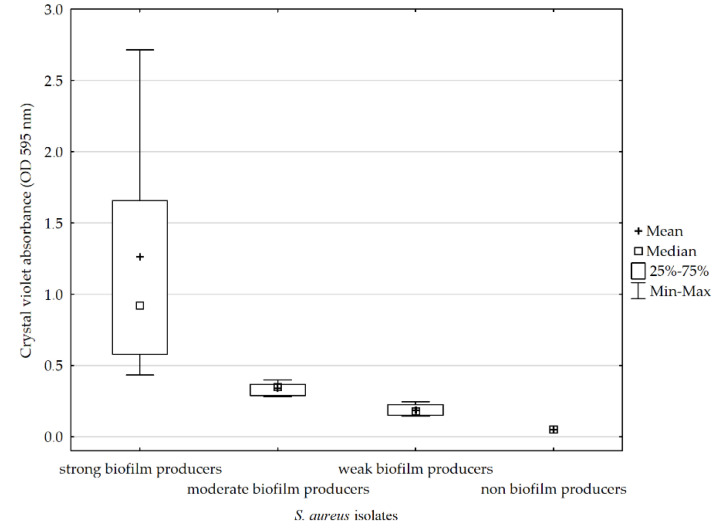
Biofilm–forming ability of *S. aureus* strains isolated from tracheostomy tubes.

The quantitative assessment of the biofilm showed that most of the strains tested (96%, n = 23) produced biofilm, and among the tested isolates, 54% (n = 13) were characterized by high biofilm production, 25% (n = 6) were classified as medium, and 17% (n = 4) as poor biofilm producers. One strain (4%) did not form a biofilm (Figure 3).

### 3.6. Phenotypic Characterization of Slime Producing Ability on CRA

**Figure 4:**
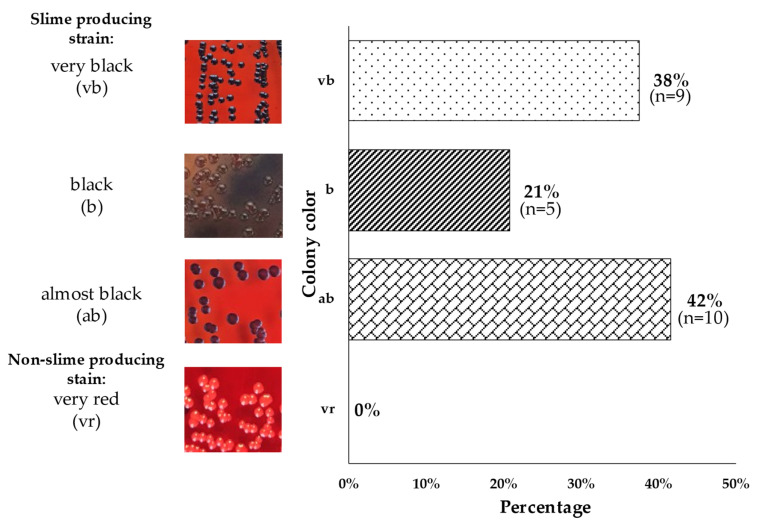
Mucus production results for *S. aureus* isolated from tracheostomy tubes using the CRA method. Legend: vb—*S. epidermidis* ATCC^®^ 35984™ (positive control); b—isolate TT_28 (1); ab—isolate TT_17; vr—*S. epidermidis* reference strain ATCC^®^ 12228™ (negative control).

A positive result of the CRA test showed that all tested strains (100%, n = 24) produced slime to a different degree, 38% (n = 9) formed very black (vb) colonies, 21% (n = 5) produced black colonies (b) and 42% (n = 10) formed almost black (ab) colonies (Figure 4).

### 3.7. MSCRAMMs

**Figure 5:**
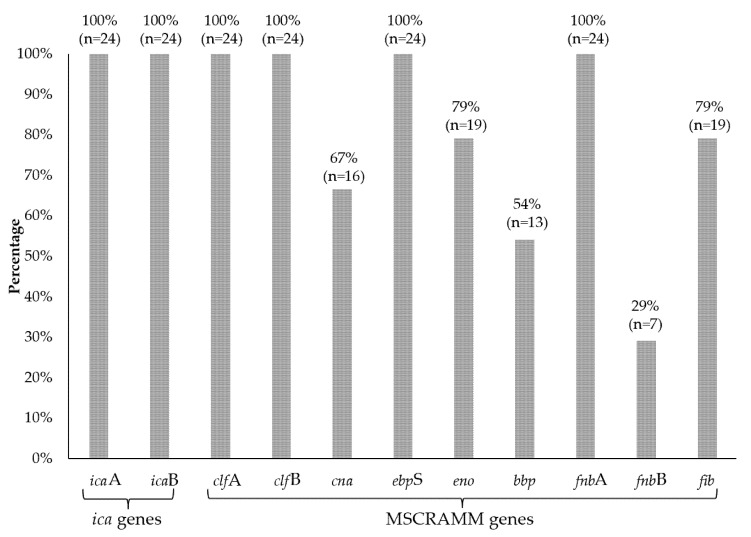
The frequency of occurrence of *ica* operon genes and genes encoding proteins from the MSCRAMM family in the studied *S. aureus* strains.

**Figure 6:**
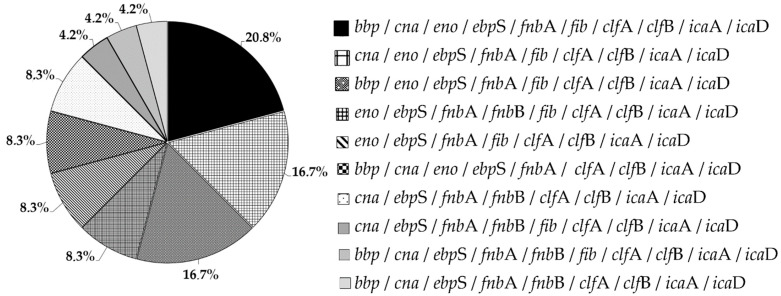
The frequency of combination of *ica* operon genes and genes encoding proteins from the MSCRAMM family in the studied *S. aureus* strains.

In the studied group of isolates, the most common genes were those encoding the bound coagulase (*clf*A and *clf*B), *fnb*A (encoding fibronectin binding protein A), *fnb*B (encoding fibronectin binding protein B) and elastin binding protein (*ebp*S), which were detected in all 100% of the strains (n = 24). In contrast, the gene encoding laminin (*eno*) and fibrinogen binding protein (*fib*) were present in 79% (n = 19) and the gene encoding collagen binding protein (*cna*) in 67% (n = 16) of the specimens. The bone sialoprotein binding protein (*bbp*) gene was detected in 54% of the isolates (n = 13) (Figure 5). The most common characteristic genotype was *bbp*/*cna*/*eno*/*ebp*S/*fnb*A/*fib*/*clf*A/*clf*B/*ica*A/*ica*D, observed in 20.8% (n = 5) of isolates, followed by *cna*/*eno*/*ebp*S/*fnb*A/*fib*/*clf*A/*clf*B/*ica*A/*ica*D and *bbp*/*eno*/*ebp*S/*fnb*A/*fib*/*clf*A/*clf*B/*ica*A/*ica*D genotypes occurring in 16.8% (n = 4) of isolates. The genotypes *eno*/*ebp*S/*fnb*A/*fnb*B/*fib*/*clf*A/*clf*B/*ica*A/*ica*D, *eno*/*ebp*S/*fnb*A/*fib*/*clf*A/*clf*B/*ica*A/*ica*D, *bbp*/*cna*/*eno*/*ebp*S/*fnb*A/*clf*A/*clf*B/*ica*A/*ica*D and *cna*/*ebp*S/*fnb*A/*fnb*B/*clf*A/*clf*B/*ica*A/*ica*D were detected in 8.3% (n = 2) of the tested strains. The least common genotypes were *cna*/*ebp*S/*fnb*A/*fnb*B/*fib*/*clf*A/*clf*B/*ica*A/*ica*D, *bbp*/*cna*/*ebp*S/*fnb*A/*fnb*B/*fib*/*clf*A/*clf*B/*ica*A/*ica*D, *bbp*/*cna*/*ebp*S/*fnb*A/*fnb*B/*clf*A/*clf*B/*ica*A/*ica*D, which were found in one isolate (4.2%) (Figure 6). A detailed analysis of individual genotypes was performed for TT-derived strains from three patients colonized simultaneously with two *S. aureus* clones, including patient no. 23 (strain no TT_23 [1] and TT_23 [2]); patient no. 28 (strain no. TT_28 [1] and TT_28 [2]); and patient no. 31 (strain no. TT_31 [1] and TT_31 [2]). For patient no. 23, the tested isolates did not differ in genotype (no. TT_23 [1] and TT_23 [2]), and similarly, for patient no. 31, the isolates (no. TT_31 [1] and TT_31 [2]) had an identical genotype. The only difference involving the genotype was the presence of the *bbp* gene in the isolates (no. TT_28 [1] and TT_28 [2]) originating from patient no. 28 (Figure 2).

### 3.8. icaAD Genes

By amplifying genes A and D from the *ica* operon, it was found that 100% (n = 24) of the tested strains had both genes tested (Figure 4).

### 3.9. Scanning Electron Microscopy (SEM)

**Figure 7:**
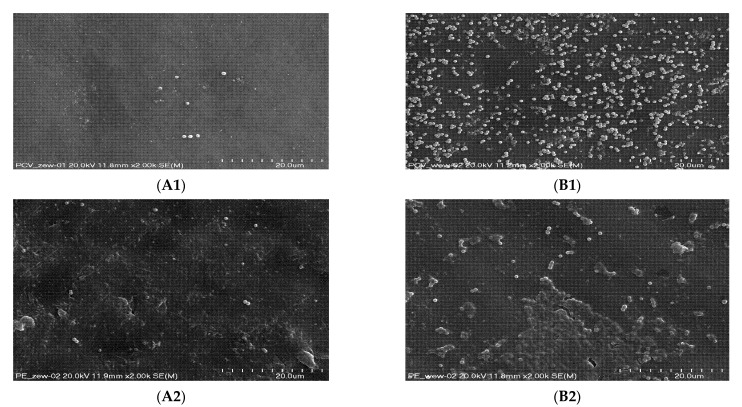
*S. aureus* microcolonies and biofilm exposed on the surface of a tracheostomy tube made of PVC (**1**) and PE (**2**) on the outer (**A**) and inner (**B**) sides. Magnification (**A1,A2,B1,B2**): 2000×.

The formed biofilm was observed using a scanning electron microscope (Figure 7). The study confirmed the ability of *S. aureus* to adhere to abiotic surfaces and showed diversity in the number of bacteria adhering to both biomaterial surfaces. A significantly larger number of single *S. aureus* cells was found on the inner surface of the PVC biomaterial. On the other hand, single *S. aureus* cells and bacterial biofilm were observed on the outer side of the PE. In the case of tracheostomy tubes made of PVC and PE, the presence of single bacterial cells was demonstrated on the external surfaces.

## 4. Discussion

Researchers frequently discuss the role of *S. aureus* bacterial biofilm in the course of infections associated with the use of medical devices made of biomaterials [6,11,27].

The ability of staphylococci to produce biofilm significantly increases the tolerance of the immune system of the host and is the cause of numerous human infections. In addition, biofilm structures provide protection against antimicrobial agents and significantly reduce the sensitivity to antibiotics; therefore, the applied antibiotic therapy becomes less effective [28,29].

The results presented and discussed below were collected and considered in terms of assessing the biofilm formation capacity of *S. aureus* isolates derived from tracheostomy tubes from hospitalized adult tracheostomy patients. The research was carried out using both phenotypic and genotypic methods.

Typing *S. aureus* with PFGE is widely used for the purposes of epidemiological analysis [30]. On the basis of the PFGE results obtained during this study, a large genetic diversity of *S. aureus* strains was found. In the studied group, only one clonal line was observed among the MRSA and MSSA strains. The results obtained in our study are very difficult to compare with those of other authors because, to the best of our knowledge, no one has ever genotyped *S. aureus* strains isolated from tracheostomy tube swabs obtained from patients after tracheostomy.

The results of drug resistance studies showed that only 8% of the tested *S. aureus* strains had the MRSA phenotype. Moreover, 50% of the tested strains showed resistance to chloramphenicol, 25% to erythromycin, 17% to tetracycline, 8% to clindamycin and 4% to norfloxacin, ciprofloxacin, moxifloxacin and ofloxacin. In contrast, the group of Jain et al. tested 79 *S. aureus* isolates, 63.29% of which were MRSA strains. The isolates came from infections related to orthopedic implants. The study population showed high resistance to erythromycin (82%), levofloxacin (72%), clindamycin and gentamicin (62%), trimethoprim-sulfamethoxazole (40%), amikacin (20%) and no vancomycin-resistant strains were found [31]. On the other hand, the team of Zariza et al. surveyed a student population for carriers of *S. aureus* in the nose. A total of 15% of the isolated strains showed the MRSA phenotype. A total of 20% were resistant to erythromycin, and none were resistant to moxifloxacin, vancomycin, or chloramphenicol [32]. The above findings demonstrate that the strains analyzed in this study exhibited high resistance to antibiotics from the groups of macrolides, lincosamides and streptogramins, tetracyclines and other antibiotics such as chloramphenicol. Resistance to macrolides, lincosamides and streptogramin B (phenotype MLS_B_) is caused by methylation of the target site of action and leads to cross-resistance of bacteria to all antibiotics in this class. We distinguish two MLS_B_ mechanisms. The inducible one (iMLS_B_) can be encoded by small multi-copy plasmids and in the chromosomal DNA of bacteria, while the constitutive MLS_B_ (cMLS_B_) type is plasmid-encoded. Chloramphenicol resistance is most often due to the activity of chloramphenicol acetyltransferase (CAT). It is encoded by a group of small multicopy plasmids. Plasmid transfer of resistance genes in *S. aureus* greatly facilitates transmission to other strains [33]. However, it should be noted that the limited number of isolates tested is not representative of the entire population; therefore, the strains tested are a limitation in estimating the percentage of resistance to the antibiotics tested.

In the crystal violet method, the obtained results showed that 96% of the strains formed a biofilm. The obtained results differ to some extent from the results of studies published in 2006 by Mathur et al., which showed that, among the tested *Staphylococcus* spp. strains isolated from blood, medical devices and skin surface, 14.47% of isolates formed large biofilm, 39.4% average, and 46% of the strains showed little or no biofilm production [34]. Furthermore, the team of Piechota et al. examined a collection of clinical isolates of *S. aureus* obtained from swabs of wounds, nose, anus, throat, tracheostomy tubes, catheters, blood, urine, abscesses, bronchoalveolar lavage and sputum, among which 36.9% were strong, 49.2% medium and 13.1% poor biofilm producers. In that study, three strains were also isolated from tracheostomy tubes. They were able to produce strong (66.7%) and moderate (33.3%) biofilms [11].

The results obtained from the CRA test showed a higher proportion of strains capable of producing mucus (100%) than the results of the experiment carried out by Arciola et al. in which 57.5% of the isolates produced mucus [24]. A positive test result for 45.5% was obtained by Nasr et al. [35]. The discrepancies may be due to the source of the isolated strains. In this study, all isolates came from tracheostomy tubes, while Nasr et al. isolated *S. aureus* from vascular catheters and from blood. In contrast, the group of Arciola et al. isolated *S. aureus* from orthopedic devices. Moreover, it should be emphasized that the CRA method is burdened with a certain error related to the subjective assessment of the color by the experimenter and the large color differentiation by bacterial colonies; therefore, the obtained results may be difficult to compare between the centers.

MSCRAMM adhesins are the main factors responsible for the formation of bacterial biofilm and play an important role in the pathogenicity of *S. aureus* strains. However, the role of these proteins in the pathogenesis of staphylococcal infections in patients with tracheostomy is still little known; therefore, in this study, research was undertaken to detect genes coding for them. There was a large variation in the frequency of adhesin-coding genes among the tested *S. aureus*. In the studied pool of strains, the *clf*A and *clf*B genes were characterized by the highest percentage of occurrence and were detected in all strains. The results of the research team led by Ghasemian are consistent with the obtained results because they also detected the presence of *clf*A and *clf*B genes in all tested strains isolated from hospitalized children with systemic infections [27]. The clfA and clfB proteins have been well studied and many of their functions have been detected in the pathogenesis of *S. aureus*. They participate in endocarditis, bacteremia or pyonephrosis [28]. Additionally, clfB is a surface protein that plays an important role in the colonization of the nasal cavity. This is due to its high affinity for cytokeratin 10, which occurs in keratinocytes (basal epithelium) [36]. Animal models also confirm that clf proteins are a virulence factor in endocarditis [28]. Moreover, the *eno* (96%), *ebp*S (83%) and *cna* (67%) genes were most often detected in the studied pool of strains. Subsequently, the *cna*, *eno* and *ebp*S genes were characterized by a frequency of 70%. In the work of Galant et al., the genes *eno*, *ebp*S and *cna* were detected in 52%, 26.4% and 16.4% of *S. aureus* isolates isolated from patients with chronic osteomyelitis, respectively [37]. On the other hand, the study by Ghasemian et al. showed the presence of *eno*, *cna* and *ebp*S in 82%, 63% and 9% of the strains, respectively [27]. The protein encoded by the *cna* gene plays an important role in keratitis in an animal model [38]. Moreover, along with *clf*A, it is involved in joint infections and bacteremia [39]. The eno protein allows it to bind to laminin, which is the main component of the basal membrane of blood vessels, which facilitates adherence to the walls of blood vessels and may contribute to the invasion of other tissues and organs [40]. Moreover, the *ebp*S gene encodes an elastin binding protein, which is an important component of the elastic fiber of the extracellular matrix [41]. The combination of the *eno* and *ebp*S genes may contribute to the high human tissue colonization capacity. All strains showed the presence of the *fnb*A gene and 29.2% strains had the *fnb*B gene. However, the *fib* gene was detected in 79% of the strains, which is partially similar to Ghasemian et al., who confirmed the presence of the genes *fnb*A in 63%, *fnb*B in 6% and *fib* in 50% of the examined *S. aureus* isolates [27]. The most common genotype was *bbp*/*cna*/*eno*/*ebp*S/*fnb*A/*fib*/*clf*A/*clf*B/*ica*A/*ica*D, which occurs in 21% of the tested isolates and contained the complete set of genes detected.

The A and D genes from the *ica* operon were detected in all the strains characterized, indicating that these strains are capable of synthesizing polysaccharide intercellular adhesin (PIA). The team of Piechota et al. tested clinical isolates of *S. aureus* for the detection of genes A and D from the *ica* operon, 98% of which had *ica*A and 96% had *ica*D [11]. In contrast, in the studies of Nasr et al., the frequency of *ica*AD detection was 32% among 50 tested isolates from intravascular catheters and blood [35]. In comparison, Nourbakhsh et al. detected the above-mentioned genes in *S. aureus,* with the frequency of *ica*A at 34.2% and *ica*D at 54.8%. The researchers isolated the tested strains from blood, bedsores, wounds, abscesses, tracheal secretions, catheters, synovial fluid, and cerebrospinal fluid [42]. However, one of the tested strains, despite the presence of the *ica*AD genes, did not produce a biofilm. This is consistent with the literature reports, which indicate that despite the presence of genes in the *ica* locus, they are not able to produce biofilm. The mechanism of the test is not fully understood [43,44,45].

The conducted research provides new information on *S. aureus* colonizing tracheostomy tubes. The detection of genes from the MSCRAMM family could help to understand the most common proteins on the surface of bacteria, and thus lead to the development of new biomaterials to prevent or limit bacterial infections associated with biomaterials.

## 5. Conclusions

It has been shown that *S. aureus* found on the surface of tracheostomy tubes shows a large variety of clonal strains, high biofilm production capacity and high resistance to certain groups of antibiotics. The present results suggest that these bacteria may come from the endogenous flora of patients. At the same time, the high frequency of occurrence of some genes encoding surface proteins from the MSCRAMM family makes the strains capable of colonizing a wide spectrum of tissues in hospitalized patients. However, no relationship was found between the high biofilm formation capacity of the tested strains and the incidence of surface proteins from the MSCRAMM family. The actual role of these genes in the pathogenesis of respiratory staphylococcal infection in patients undergoing tracheostomy remains largely unknown.

## Figures and Tables

**Table 1:** Primer sequences used in the study.

No.	Species/Gene	Sequence (5′ → 3′)	Product Size (pz)	Source
**Molecular confirmation of the species *Staphylococcus aureus* and detection of *mec*A gene**				
1	*S. aureus*	AATCTTTGTCGGTACACGATATTCTTCACG	108	[18]
		CGTAATGAGATTTCAGTAGATAATACAACA		
2	*S. epidermidis*	ATCAAAAAGTTGGCGAACCTTTTCA	124	
		CAAAAGAGCGTGGAGAAAAGTATCA		
3	*S. haemolyticus*	GGTCGCTTAGTCGGAACAAT	271	
		CACGAGCAATCTCATCACCT		
4	*mec*A	TAGAAATGACTGAACGTCCG	154	
		TTGCGATCAATGTTACCGTAG		
**Operon *ica***				
5	*ica*A	ACACTTGCTGGCGCAGTCAA	188	[11]
		TCTGGAACCAACATCCAACA		
6	*ica*D	ATGGTCAAGCCCAGACAGAG	198	
		AGTATTTTCAATGTTTAAAGCAA		
**MSCRAMM**				
7	*bbp*	AACTACATCTAGTACTCAACAACAG	575	[25]
		ATGTGCTTGAATAACACCATCATCT		
8	*cna*	GTCAAGCAGTTATTAACACCAGAC	423	
		AATCAGTAATTGCACTTTGTCCACTG		
9	*eno*	ACGTGCAGCAGCTGACT	302	
		CAACAGCATYCTTCAGTACCTTC		
10	*ebp*S	CATCCAGAACCAATCGAAGAC	186	
		CTTAACAGTTACATCATCATGTTTATCTTTG		
11	*fnb*A	GTGAAGTTTTAGAAGGTGGAAAGATTAG	643	
		GCTCTTGTAAGACCATTTTTCTTCAC		
12	*fnb*B	GTAACAGCTAATGGTCGAATTGATACT	524	
		CAAGTTCGATAGGAGTACTATGTTC		
13	*fib*	CTACAACTACAATTGCCGTCAACAG	404	
		GCTCTTGTAAGACCATTTTCTTCAC		
14	*clf*A	ATTGGCGTGGCTTCAGTGCT	292	
		CGTTTCTTCCGTAGTTGCATTTG		
15	*clf*B	ACATCAGTAATAGTAGGGGGCAAC	205	
		TTCGCACTGTTTGTGTTTGCAC		

## Data Availability

Data sharing not applicable.

## References

[1] Laux C., Peschel A., Krismer B. (2019). *Staphylococcus aureus* Colonization of the Human Nose and Interaction with Other Microbiome Members. Microbiol. Spectr..

[2] Lowy F.D. (1998). *Staphylococcus aureus* infections. N. Engl. J. Med..

[3] Tong S.Y.C., Davis J.S., Eichenberger E., Holland T.L., Fowler V.G. (2015). *Staphylococcus aureus* Infections: Epidemiology, Pathophysiology, Clinical Manifestations, and Management. Clin. Microbiol. Rev..

[4] Rezende-Pereira G., Albuquerque J.P., Souza M.C., Nogueira B.A., Silva M.G., Hirata R., Mattos-Guaraldi A.L., Duarte R.S., Neves F.P.G. (2021). Biofilm Formation on Breast Implant Surfaces by Major Gram-Positive Bacterial Pathogens. Aesthetic Surg. J..

[5] Hasan Abdul Cader S., Fahim Ahmed Shah F.A. (2020). Tracheostomy colonisation and microbiological isolates of patients in intensive care units-a retrospective study. World J. Otorhinolaryngol. Head Neck Surg..

[6] Arciola C.R., Campoccia D., Montanaro L. (2018). Implant infections: Adhesion, biofilm formation and immune evasion. Nat. Rev. Microbiol..

[7] El Cheik M.R., Barbosa J.M., Caixêta J.A.S., Avelino M.A.G. (2018). Microbiology of Tracheal Secretions: What to Expect with Children and Adolescents With Tracheostomies. Int. Arch. Otorhinolaryngol..

[8] Abdollahi A., Shoar S., Shoar N. (2013). Microorganisms’ colonization and their antibiotic resistance pattern in oro—tracheal tube. Iran. J. Microbiol..

[9] Lepainteur M., Ogna A., Clair B., Dinh A., Tarragon C., Prigent H., Davido B., Barbot F., Vaugier I., Afif M. (2019). Risk Factors for Respiratory Tract Bacterial Colonization in Adults with Neuromuscular or Neurological Disorders and Chronic Tracheostomy. Respir. Med..

[10] Bjarnsholt T., Jensen P.Ø., Fiandaca M.J., Pedersen J., Hansen C.R., Andersen C.B., Pressler T., Givskov M., Høiby N. (2009). *Pseudomonas aeruginosa* biofilms in the respiratory tract of cystic fibrosis patients. Pediatric Pulmonol..

[11] Piechota M., Kot B., Frankowska-Maciejewska A., Gruzewska A., Woźniak-Kosek A. (2018). Biofilm Formation by Methicillin-Resistant and Methicillin-Sensitive *Staphylococcus aureus* Strains from Hospitalized Patients in Poland. BioMed Res. Int..

[12] Paharik A.E., Horswill A.R. (2016). The *Staphylococcal* Biofilm: Adhesins, Regulation, and Host Response. Microbiol. Spectr..

[13] Lister J.L., Horswill A.R. (2014). *Staphylococcus aureus* biofilms: Recent developments in biofilm dispersal. Front. Cell. Infect. Microbiol..

[14] Folliero V., Franci G., Dell’Annunziata F., Giugliano R., Foglia F., Sperlongano R., De Filippis A., Finamore E. (2021). Evaluation of Antibiotic Resistance and Biofilm Production among Clinical Strain Isolated from Medical Devices. Int. J. Microbiol..

[15] Cucarella C., Solano C., Valle J., Amorena B., Lasa I., Penadés J.R. (2001). Bap, a *Staphylococcus aureus* Surface Protein Involved in Biofilm Formation. J. Bacteriol..

[16] Götz F. (2002). *Staphylococcus* and biofilms. Mol. Microbiol..

[17] Arciola C.R., Baldassarri L., Montanaro L. (2001). Presence of *ica*A and *ica*DGenes and Slime Production in a Collection of *Staphylococcal* Strains from Catheter-Associated Infections. J. Clin. Microbiol..

[18] Pereiraab E.M., Schuencka E.P., Malvara K.L., Iorioa N.L.P., Matosa P.D.M., Olendzkic A.N., Oelemanna W.M.R., dos Santosa K.R.N. (2010). *Staphylococcus aureus*, *Staphylococcus epidermidis* and *Staphylococcus haemolyticus*: Methicillin-resistant isolates are detected directly in blood cultures by multiplex PCR. Microbiol. Res..

[19] EUCAST European Committee on Antimicrobial Susceptibility Testing Breakpoint tables for Interpretation of MICs and Zone Diameters Version 11.0, Valid from 2021-01-01. http://www.eucast.org.

[20] Murchan S., Kaufmann M.E., Deplano A., de Ryck R., Struelens M., Zinn C.E., Fussing V., Salmenlinna S., Vuopio-Varkila J., El Solh N. (2003). Harmonization of Pulsed-Field Gel Electrophoresis Protocols for Epidemiological Typing of Strains of Methicillin-Resistant *Staphylococcus aureus*: A single approach developed by consensus in 10 European laboratories and its application for tracing the spread of related strains. J. Clin. Microbiol..

[21] Ochońska D., Ścibik Ł., Brzychczy-Włoch M. (2021). Biofilm Formation of Clinical *Klebsiella pneumoniae* Strains Isolated from Tracheostomy Tubes and Their Association with Antimicrobial Resistance, Virulence and Genetic Diversity. Pathogens.

[22] Stepanović S., Vuković D., Hola V., Di Bonaventura G., Djukić S., Cirković I., Ruzicka F. (2007). Quantification of biofilm in microtiter plates: Overview of testing conditions and practical recommendations for assessment of biofilm production by staphylococci. Acta Pathol. Microbiol. Scand..

[23] Freeman D.J., Falkiner F.R., Keane C.T. (1989). New method for detecting slime production by coagulase negative *staphylococci*. J. Clin. Pathol..

[24] Arciola C.R., Campoccia D., Gamberini S., Cervellati M., Donati E., Montanaro L. (2002). Detection of slime production by means of an optimised Congo Red agar plate test based on a colourimetric scale in *Staphylococcus epidermidis* clinical isolates genotyped for *ica* locus. Biomaterials.

[25] Tristan A., Ying L., Bes M., Etienne J., Vandenesch F., Lina G. (2003). Use of multiplex PCR to identify *Staphylococcus aureus* adhesins involved in human hematogenous infections. J. Clin. Microbiol..

[26] Pajerski W., Duch J., Ochonska D., Gołda-Cępa M., Brzychczy-Włoch M., Kotarba A. (2020). Bacterial attachment to oxygen-functionalized graphenic surfaces. Mater. Sci. Eng. C.

[27] Ghasemian A., Peerayeh S.N., Bakhshi B., Mirzaee M. (2015). The Microbial Surface Components Recognizing Adhesive Matrix Molecules (MSCRAMMs) Genes among Clinical Isolates of *Staphylococcus aureus* from Hospitalized Children. Iran. J. Pathol..

[28] Foster T.J. (2019). The MSCRAMM Family of Cell-Wall-Anchored Surface Proteins of Gram-Positive Cocci. Trends Microbiol..

[29] Mah T.F., O’Toole G.A. (2001). Mechanisms of biofilm resistance to antimicrobial agents. Trends Microbiol..

[30] Golding G.R., Campbell J., Spreitzer D., Chui L. (2015). Pulsed-field gel electrophoresis of *Staphylococcus aureus*. Methods Mol. Biol..

[31] Jain S., Chowdhury R., Datta M., Chowdhury G., Mukhopadhyay A.K. (2019). Characterization of the clonal profile of methicillin resistant *Staphylococcus aureus* isolated from patients with early post-operative orthopedic implant based infections. Ann. Clin. Microbiol. Antimicrob..

[32] Zariza S., Yeo C.C., Faizal G.M., Chew C.H., Zakaria Z.A., Al-Obaidi M.M.J., Amin N.S., Nasir M.D.M. (2018). Nasal colonisation, antimicrobial susceptibility and genotypic pattern of *Staphylococcus aureus* among agricultural biotechnology students in Besut, Terengganu, east coast of Malaysia. Trop. Med. Int. Health.

[33] Lyon B.R., Skurray R.A. (1987). Antimicrobial resistance of *Staphylococcus aureus*: Genetic basis. Microbiol. Rev..

[34] Mathur T., Singhal S., Khan S., Upadhyay D.J., Fatma T., Rattan A. (2006). Detection of biofilm formation among the clinical isolates of *Staphylococi*: An evaluation of three different screening methods. Indian J. Med. Microbiol..

[35] Nasr R.A., AbuShady H.M., Hussein H.S. (2012). Biofilm formation and presence of *ica*AD gene in clinical isolates of staphylococci. Egypt. J. Med. Hum. Genet..

[36] Wertheim H.F.L., Walsh E., Choudhurry R., Melles D.C., Boelens H.A.M., Miajlovic H., Verbrugh H.A., Foster T., van Belkum A. (2008). Key role for clumping factor B in *Staphylococcus aureus* nasal colonization of humans. PLoS Med..

[37] Galant K., Giedrys-Kalemba S., Johaniuk A., Roszkowska P., Jursa-Kulesza J. (2016). Occurence of factors associated with ability to biofilm formation among methicillin-sensitive and methicillin-resistant *Staphylococcus aureus* strains isolated from patients with bone infections. Forum Zakażeń.

[38] Rhem M.N., Lech E.M., Patti J.N., McDevitt D., Höök M., Jones D.B., Wilhelmus K.R. (2000). The collagen-binding adhesin is a virulence factor in *Staphylococcus aureus* keratitis. Infect. Immun..

[39] Josefsson E., Hartford O., O’Brien L., Patti J.M., Foster T. (2001). Protection against experimental *Staphylococcus aureus* arthritis by vaccination with clumping factor A, a novel virulence determinant. J. Infect. Dis..

[40] Carneiro C.R.W., Postol E., Nomizo R., Reis L.F.L., Brentani R.R. (2004). Identification of enolase as a laminin-binding protein on the Surface of *Staphylococcus aureus*. Microbes Infect..

[41] Downer R., Roche F., Park P.W., Mecham R.P., Foster T.J. (2002). The Elastin-binding Protein of *Staphylococcus aureus* (EbpS) Is Expressed at the Cell Surface as an Integral Membrane Protein and Not as a Cell Wall-associated Protein. J. Biol. Chem..

[42] Nourbakhsh F., Namvar A.E. (2016). Detection of genes involved in biofilm formation in *Staphylococcus aureus* isolates. GMS Hyg. Infect. Control.

[43] Grinholc M., Wegrzyn G., Kurlenda J. (2007). Evaluation of biofilm production and prevalence of the *ica*D gene in methicillin-resistant and methicillin-susceptible *Staphylococcus aureus* strains isolated from patients with nosocomial infections and carriers. FEMS Immunol. Med. Microbiol..

[44] Cramton S.E., Gerke C., Schnell N.F., Nichols W.W., Gotz F. (1999). The intercellular adhesion (*ica*) locus is present in *Staphylococcus aureus* and is required for biofilm formation. Infect. Immun..

[45] Fowler V.G., Fey P.D., Reller L.B., Chamis A.L., Corey G.R., Rupp M.E. (2001). The intercellular adhesin locus *ica* is present in clinical isolates of *Staphylococcus aureus* from bacteremic patients with infected and uninfected prosthetic joints. Med. Microbiol. Immunol..

